# Designing Multi-Epitope Vaccines to Combat Emerging Coronavirus Disease 2019 (COVID-19) by Employing Immuno-Informatics Approach

**DOI:** 10.3389/fimmu.2020.01663

**Published:** 2020-07-10

**Authors:** Anam Naz, Fatima Shahid, Tariq Tahir Butt, Faryal Mehwish Awan, Amjad Ali, Arif Malik

**Affiliations:** ^1^Institute of Molecular Biology and Biotechnology (IMBB), The University of Lahore (UOL), Lahore, Pakistan; ^2^Atta-ur-Rehman School of Applied Biosciences (ASAB), National University of Sciences and Technology (NUST), Islamabad, Pakistan; ^3^Department of Biochemistry, Khawaja Muhammad Safdar Medical College, Sialkot, Pakistan

**Keywords:** COVID-19, coronavirus, corona vaccine, spike protein, S1 domain, S2 domain

## Abstract

A recent pandemic caused by a single-stranded RNA virus, COVID-19, initially discovered in China, is now spreading globally. This poses a serious threat that needs to be addressed immediately. Genome analysis of SARS-CoV-2 has revealed its close relation to SARS-coronavirus along with few changes in its spike protein. The spike protein aids in receptor binding and viral entry within the host and therefore represents a potential target for vaccine and therapeutic development. In the current study, the spike protein of SARS-CoV-2 was explored for potential immunogenic epitopes to design multi-epitope vaccine constructs. The S1 and S2 domains of spike proteins were analyzed, and two vaccine constructs were prioritized with T-cell and B-cell epitopes. We adapted a comprehensive predictive framework to provide novel insights into immunogenic epitopes of spike proteins, which can further be evaluated as potential vaccine candidates against COVID-19. Prioritized epitopes were then modeled using linkers and adjuvants, and respective 3D models were constructed to evaluate their physiochemical properties and their possible interactions with ACE2, HLA Superfamily alleles, TLR2, and TLR4.

## Introduction

A rapid increase in the human population and its mobility has led to urbanization and subsequent climate and ecological changes, catering to emerging infectious diseases that galvanize an implacable threat to human health around the world (1). The human race has encountered multiple bacterial and viral pathogens, some being inconsequential while others causing global chaos. Interestingly, before the twenty-first century, human coronaviruses were thought to be trivially harmful, causing only common cold in healthy individuals (2).

Coronaviruses have an enveloped positive-sense RNA genome comprising about 25–32 kilobases. They have been identified in multiple mammalian hosts, including dogs, cats, bats, camels, pigs, and civets (3). According to Centers for Disease Control and Prevention (CDC), common human infecting coronaviruses include 229E coronavirus, NL63 coronavirus, OC43 beta coronavirus, HKU1 coronavirus, MERS-CoV, SARS-CoV, and the recently emerged deadly coronavirus disease 2019 (COVID-19). The first four account for 10–30% of upper respiratory tract infections in human adults. While the latter three have emerged as perpetual challenge for the scientific community.

In November 2002, an outbreak of severe acute respiratory syndrome coronavirus (SARS-CoV) in Guangdong, China, led to the deaths of around 774 out of ~8,000 infected individuals from 37 different countries (4). Common symptoms in SARS-infected individuals were documented as cough, fever, dyspnea, and occasional diarrhea. Although sequence analysis of the virus depicted that bats were its hosts, human-to-human transmission was also observed (5, 6). Likewise, in 2012, the emergence of Middle East respiratory syndrome coronavirus (MERS-CoV) was reported in Saudi Arabia (7). The symptoms included atypical pneumonia along with gastrointestinal problems and kidney failure. As a result, out of 2,494 reported cases, 858 patients have died to date as of November 2019 (World Health Organization report).

In December 2019, COVID-19 was initially encountered in Wuhan, China, and has now rapidly spread to multiple countries. The affected individuals exhibit mild symptoms that turn into pneumonia as the illness progresses (8). According to nature news, as of February 7th, this virus is responsible for infecting about 31,161 humans in China, leading to the death of 630 patients. The majority of the cases tend to have some connection to the seafood and animal market, which indicates the virus is zoologically transmitted. This situation has gained the attention of authorities at both a local and state level and has highlighted an urgent need to devise a method for rapid treatment of the deadly pathogen (9, 10).

Recent research has established that the RNA genome of recently discovered SARS-CoV2 comprises of 9,860 amino acids. It features two untranslated regions at both flanking ends while only a single polyprotein encoding open reading frame is present between them. The genome is organized in a sequential manner starting from 5' replicase, and it is followed by structural proteins: the spike, envelope, and nucleocapsid at the *N* terminal (11). Reportedly, the spike protein acts as multifunctional molecular machinery to mediate viral entry into host cells and is involved in viral transmission. Initially, it binds the host cell-surface receptor via the S1 subunit domain and afterwards carries out the fusion of host and viral cell membranes with the help of the S2 domain. A wide variety of host receptors can be recognized by two subsequent domains in S1 region of SARS-CoV-2, leading to viral attachment. The N-terminal peptide domain (ranges from amino acid 14–305 in the sequence) as well as the C-terminal peptide domain (the receptor binding domain ranging from amino acid number 319 to 541) of the S1 zone have the ability to bind host cell receptors. It has been suggested that SARS-CoV-2 exploits angiotensin-converting enzyme 2 (ACE2) as a cell receptor (10, 12, 13).

Outbreaks of infectious disease like COVID-19 poses a serious challenge to the scientific community since they usually arise from unrecognized zoonotic sources or due to scarcity data. Viruses can emerge by evolving from their animal-restricted form to another form that can infect humans by attainment of their receptors and biosynthetic machinery. A majority of the recently emerging pathogens are difficult to treat due to the lack of specific therapeutic options (14). So far, no therapeutic vaccine for either SARS-CoV, MERS-CoV, or SARS-CoV-2 currently exists in the market, although some clinical trials are in progress (15).

Innovative computational biology approaches have enabled us to obtain immunogenic and highly conserved epitopes from bacterial and viral antigens (16–19). Both CD4^+^ and CD8^+^ epitopes can be used separately or in combination to construct broad spectrum vaccine candidates. The proposed vaccines can combat a wide variety of pathogens and possess the ability to elicit cellular and humoral responses in human hosts. Once administered, the mock epitopes from the vaccine are presented by MHC. The presented epitopes are recognized by their corresponding T-cell receptors that proliferates and generates suitable immune responses. Considering this, T-cell epitopes from deadly pathogens can facilitate T-cell-based vaccine development (CD4^+^ and CD8^+)^. More precisely, a CD4^+^-based subunit vaccine usually deals with exogenous antigens that are phagocytosed by APCs and subsequently bind to MHC-II, which presents them to CD4^+^ T cells. Accordingly, a CD8^+^-based T-cell vaccine encompasses endogenous antigens that are degraded by APCs and later presented via MHC-I to CD8^+^ T cells (17, 19, 20).

Epitope-based chimeric/subunit vaccines have many advantages when compared to vaccines produced via conventional vaccinology. For instance, they are cheaper to develop, do not require microbial culturing, and can surpass many wet lab experiments, saving time. They are a safer option, as they do not contain the entire pathogen and are highly specific and stable (21). Nevertheless, due to the presence of mutable HLA variants, epitope-based vaccines targeting limited HLA alleles usually do not produce the required/equal effect among the human population. Hence highly promiscuous epitopes can bind multiple alleles at a time and can ensure the desired immune response among a heterogeneous human population (18). The current study focuses on finding promiscuous CD4^+^ and CD8 T^+^ cell epitopes for chimeric COVID-19 vaccine development using a variety of web-based tools. The proposed potential vaccine is then checked for its binding affinity with suitable receptors.

## Materials and Methods

### Sequence Acquisition and Prediction of T- and B-Cell Epitopes

The surface glycoprotein sequence of the pneumonia virus discovered at the Wuhan seafood market (QHD43416.1 from MN908947.3 reference genome) was retrieved from NCBI (22). To scrutinize required HLA binding epitopes, a TepiTool from IEDB was used (23). A set of 12 MHC class I super-types (A^*^01:01, A^*^02:01,A^*^03:01, A^*^24:02,A^*^26:01, B^*^07:02, B^*^08:01, B^*^27:05, B^*^39:01, B^*^40:01, B^*^58:01, and B^*^15:01) were used, and the two highest-scoring epitopes (based on percentile rank and IC50 values) for each allele were selected. A percentile rank is calculated by the comparison of the peptide's predicted binding-affinity against a panel of a variety of peptides randomly selected from the Swiss-Prot. Hence, a lower percentile rank numerical value depicts better binders. Additionally, all the predicted peptides were checked for their IC50 value, and those with IC50 ≤ 500 nM were taken into account. Specific immune responses are based on CD4^+^ and CD8^+^ T cells, and protective vaccines should thus induce specific T-cell responses based on peptides represented by MHC-I and MHC-II alleles. The rationale behind prioritizing HLA binding epitopes is to ensure the specific immune response in infected macrophages.

For MHC-II-binding peptide epitopes, the seven-allele method was used. This selection is based on the median of consensus percentile ranks among the seven commonly encountered DR alleles, namely, HLA-DRB1^*^03:01, HLA-DRB1^*^07:01, HLA-DRB1^*^15:01, HLA-DRB3^*^01:01, HLA-DRB3^*^02:02, HLA-DRB4^*^01:01, and HLA-DRB5^*^01:01. Epitopes with a median consensus percentile rank ≤ 20.0 were designated as good binders. The scrutiny of promiscuous peptide epitopes was established based on the median of the consensus percentile rank of the seven preselected alleles.

For B-cell epitope prediction, BepiPred 2.0 from Immune Epitope Database Analysis Resource (IEDB-AR) was used (24). IEDB-AR is linked to IEDB and offers computational analysis regarding both B and T cell epitope prediction and their subsequent analysis. BepiPred 2.0 works on the basis of a randomly chosen forest algorithm that has been trained on epitopes acquired from antibody–antigen models obtained from interactive protein structures (25).

### Epitope Screening

Owing to the significance of spike protein, the selected epitopes were manually screened for their presence in this zone. The epitopes were further examined for antigenic potential via VaxiJen version 2.0 (26). A threshold value of 0.5 was taken into account. Non-antigenic peptides (having VaxiJen score <0.5) were discarded, while antigenic epitopes (with threshold value > 0.5) were further prioritized for their immunogenicity. The Immune epitope database (IEDB) tool for immunogenicity score calculation was used to predict immunogenicity scores for all MHC-I predicted epitopes (27). This tool is designed to predict immunogenicity of the peptide based on amino-acid position and properties. Immunogenic epitopes were then verified for their presence in IEDB database.

### Construction of Chimeric Vaccine(s)

Shortlisted top-scoring epitopes were checked for their binding affinity with each other for determining the final sequence of the chimeric vaccine. The epitopes were analyzed using a HADDOCK web server (Guru interface) (28). Clusters representing two epitopes, which possessed the highest interaction scores, depicting their maximum interaction, were refined by removing the water molecules, which may hinder their interaction, and then having them dock to the third epitope. Likewise, evaluation of clusters with three epitopes was done. The refined and the highest-scoring cluster was docked to the fourth epitope to obtain the final sequence.

To facilitate the process of vaccine development, a flexible linker GGGGS was added between each epitope. This helps to restore protein folding by allowing interaction between different domains (16, 29). Additionally, another linker EAAAK was added at the *N* terminal to separate bi-functional domains. Designed vaccines were then tested with different epitopes, including Truncated Ov-ASP-1 Protein (residues 10–153) and Beta defensin (45 residues long), and constructs having higher antigenicity and that are predicted to produce high antibody titers were added with the multi epitope vaccine construct to the enhance immune response (30). Three different constructs were designed in this study, one comprising the top-scoring CD4 and CD8 epitopes lying in the S1 domain, while another is formed by taking two epitopes from the S1 domain and two from the S2 domain, representing MHC-I and MHC-II binders. Finally, the third one is formed by adding a B-cell epitope to the second one but with a different adjuvant.

### Evaluation of Physicochemical Parameters of the Chimeric Vaccine Construct

The final sequences of the chimeric vaccine constructs were screened for its antigenic potential and solubility using ANTIGENpro and SOLpro (31). Allertop version 2.0 was used to check the probability of the construct to cause an allergic reaction (32). Sequence of the finalized vaccine candidate in FASTA format was given as an input to ExPASy server, in order to calculate various parameters like molecular weight, theoretical PI, half-life of the protein, instability index, amino acid composition, aliphatic index, and GRAVY (33).

### Secondary and Tertiary Structure Prediction

Secondary structures of the vaccine constructs were predicted using PDBsum (34). This step was executed to better understand the structures of predicted vaccines. PDBsum is a database that is exclusively designed to show the molecules that build DNA or proteins, ligands, and metal ions along with the illustration of graphical representation of their interactions with each other. To generate 3D structures of the vaccine candidates, 3Dpro was used (31). The predicted models were then refined using Galaxy refine server (35). This server is responsible for subjecting the predicted 3D model to structural perturbations and subsequent structural relaxations. It generates five different models. All five models for each vaccine construct were screened for GDT-HA, RMSD, and poor rotamers, and the finest predicted models were taken to the next step.

The finalized models were further evaluated using ERRAT scores and Ramachandran plot analysis for verification. In order to obtain stabilized vaccine constructs, energy minimization was carried out using online YASARA server. YASARA deals with molecular-dynamics simulations of the given models in solvent, using an exclusive forcefield that has been derived from Amber, whose constraints have been improved to minimalize the impairment done to protein structure during the process of energy minimization (36).

### Docking Analysis

In order to study the binding affinity of the putative vaccine candidates with immune receptors, molecular docking technique was adopted. Prioritized vaccine constructs were docked to ACE2 receptor (PDB ID: 3sci), TLR2 (PDB ID: 2Z7X), and TLR4 (PDB ID: 4G8A). Vaccine 3, having a B-cell epitope, was also checked for its interaction with a B-cell receptor (BCR) CD79 (PDB ID: 3KG5). For this protein–protein docking validation process, the HAADOCK server (Guru Interface and refinement interface) was used (28). Additionally, to obtain a graphical illustration of the interactions between vaccine and receptor, PDBsum was used (34). Moreover, in order to verify the binding affinity of our multiepitope peptide vaccines with HLA alleles, all our vaccine constructs were docked with class I and class II Superfamily alleles to reveal the interaction of epitopes with MHC alleles when combined as well. Hence, for this purpose, class I [HLA A^*^02 01 (PDB ID 4U6Y), HLA B^*^51 01 (PDB ID 4MJI)] and class II [HLA-DRB1^*^1402 (PDB ID 6ATF)] were used; they represent broad-spectrum peptide-binding repertoires.

### Population Coverage Analysis

Population coverage of epitopes was determined using IEDB for prioritized epitopes, as it helps to determine the percentage population that can respond to the particular epitope and can elicit an immune response against it.

## Results

### Epitope Screening

#### Prediction of HLA Class I Binders

Initially, 15,181 HLA class I epitopes have been predicted within spike glycoprotein of COVID-19. Scrutiny on the basis of percentile rank filtered 24 peptide epitopes. Each of them had a considerable binding affinity for the 12 superfamily alleles. All of these epitopes, along with their features and respective binding alleles, are reported in Table S1. Further analysis revealed that 11 predicted epitopes lie within the S1 domain of the spike protein, eight epitopes lie in the N terminal domain (13–317 aa), and three epitopes are in the receptor-binding domain (347–520aa).

Vaxijen antigenic score prediction at a threshold of 0.5 was used to detect the antigenicity of peptide epitopes. Antigenic epitopes tend to trigger a large number of antibody titers to fight the infection. Among predicted epitopes of COVID-19 virus, six epitopes showed considerable antigenic potential, including five from the N-terminal domain and one from the receptor binding domain. An immunogenicity analysis was then carried out for further filtration, and, consequently, five epitopes were screened out; one of the epitopes lying within N-terminal domain showed relatively less immunogenicity value. Out of these five MHC-I epitopes, two epitopes from S1 domain with high antigenic and immunogenicity score were further selected for multi-epitope vaccine construction. These were ^89^GVYFASTEK^97^ and ^50^STQDLFLPF^58^. ^89^GVYFASTEK^97^ is a part of the N-terminal binding domain with antigenicity and immunogenicity scores of 0.7112 and 0.09023, respectively. Epitope ^50^STQDLFLPF^58^ also lies within the N-terminal domain and has an antigenicity and immunogenicity score of 0.6619 and 0.06828, respectively. Moreover, another HLA class I epitope ^733^KTSVDCTMY^741^ from the s2 domain of the spike proteins was also screened to be potential candidates for multi-epitope vaccine construction.

#### Prediction of HLA Class 2 Binders

A total of 1,772 unique epitopes against seven DRB alleles were identified. Twenty (15-mer epitopes) epitopes were screened out via filtration on the basis of median percentile rank <20 (Table S2). The major portion of binding energy between a peptide epitope and MHC class II receptor molecule is delivered through the basic peptide core, comprising ~9 amino acids in length. Nevertheless, the existence of extra amino acids around the basic binding core seems to play a significant role in stable binding even if they do not precisely bind the peptide-binding-groove of the MHC receptor. The 15-mer epitopes for binding with MHC-II are thus usually recommended (23).

Ten of MHC class II epitopes (three in the receptor-binding area and seven in the N-terminal domain) were found to be a part of the S1 domain. While considering a total of 10 S1 epitopes, four were found to be highly antigenic (threshold > 0.5). Among these, three belonged to the N-terminal domain of S1 while 1 was a part of the receptor-binding domain. Two epitopes ^191^EFVFKNIDGYFKIYS^205^ and ^506^QPYRVVVLSFELLHA^520^ were selected for vaccine 1 construction on the basis of their high antigenic potential. The former belonged to the N-terminal domain with an antigenicity score of 1.0339, while the later was a part of the receptor-binding domain and had an antigenicity score of 0.9109. An epitope ^731^MTKTSVDCTMYICGD^745^ from the s2 domain was also prioritized and was used along with the S1 epitope ^506^QPYRVVVLSFELLHA^520^ for vaccine 2 construction. To the best of our knowledge, none of the epitopes reported in this study have been previously added to the IEDB database. Table 1 shows the final epitopes picked for vaccine development.

**Table 1:** Finalized epitopes for vaccine constructs.

**Vaccine combination**	**Epitope**	**Representation**	**MHC class/B cell**	**Location within spike protein**	**Best binding allele**	**Percentile rank**
Vaccine 1	^89^GVYFASTEK^97^	E1S1	I	S1 domain	HLA-A*03:01	0.2
	^50^STQDLFLPF^58^	E2S1	I	S1 domain	HLA-B*15:01	0.3
	^191^ EFVFKNIDGYFKIYS^205^	E3S1	II	S1 domain	HLA-DRB5*01:01	0.17
	^506^QPYRVVVLSFELLHA^520^	E4 S1	II	S1 domain	HLA-DRB4*01:01	2.9
Vaccine 2	^89^GVYFASTEK^97^	E1 S1	I	S1 domain	HLA-A*03:01	0.2
	^733^KTSVDCTMY^741^	E1 S2	I	S2 domain	HLA-A*01:01	0.63
	^506^QPYRVVVLSFELLHA^520^	E4 S1	II	S1 domain	HLA-DRB4*01:01	2.9
	^731^MTKTSVDCTMYICGD^745^	E2 S2	II	S2 domain	HLA-DRB3*01:01	6.3
Vaccine 3	^89^GVYFASTEK^97^	E1S1	I	S1 domain	HLA-A*03:01	0.2
	^733^KTSVDCTMY^741^	E1 S2	I	S2 domain	HLA-A*01:01	0.63
	^506^QPYRVVVLSFELLHA^520^	E4 S1	II	S1 domain	HLA-DRB4*01:01	2.9
	^731^MTKTSVDCTMYICGD^745^	E2 S2	II	S2 domain	HLA-DRB3*01:01	6.3
	^369^YNSASFSTFKCYGVSPTKLNDLCFT^393^	E5 S1	B Cell	S1 domain	N/A	N/A

#### Prediction of B-Cell Epitopes

An IEDB server was used to identify 34 B cell epitopes. Out of these, 11 were found to be antigenic in nature (threshold > 0.5). They were further checked for their allergenicity, and the highly antigenic epitope, found to be non-allergenic in nature (^369^YNSASFSTFKCYGVSPTKLNDLCFT^393^), was picked. This epitope was conjugated with the S1 and S2 epitopes along with a Beta defensin adjuvant to design the vaccine 3 construct.

Envelope-affixed spike protein of coronaviruses plays an important role in receptor recognition. Several virology studies have been carried out to discover the exact mechanism of receptor binding and subsequent entry into the host cells. The SARS-CoV-2 spike protein has been found to be 76% identical to the SARS-CoV Urbani stains' spike protein and 80% identical to the bat SARSr-CoV ZXC21 and ZC45 spike protein (37). The shortlisted epitopes have also been subjected to conservation analysis, hence manifesting cross protection against other species. Conservation analysis revealed the high similarity between the prioritized epitopes of the SARS-CoV-2 spike protein with MERS and SARS spike protein epitopes (Table 2). All our seven epitopes were found to be a part of at least eight viral sequences present on NCBI, while one of the prioritized epitopes, KTSVDCTMY, was found to be 100% identical in 43 available coronavirus sequences (Table S3).

**Table 2:** Conservation analysis of prioritized epitopes from SARS-CoV-2 with SARS and MERS spike proteins.

**Identity with sequence**	**Epitope sequence**	**Epitope length**	**Percent of protein sequence matches at identity ≤ 100%**
SARS reference strain Spike protein	GVYFASTEK	9	78.57% (11/14)
	STQDLFLPF	9	92.86% (13/14)
	EFVFKNIDGYFKIYS	15	28.57% (4/14)
	QPYRVVVLSFELLHA	15	35.71% (5/14)
	KTSVDCTMY	9	78.57% (11/14)
	MTKTSVDCTMYICGD	15	21.43% (3/14)
MERS reference strain Spike protein	GVYFASTEK	9	100.00% (11/11)
	STQDLFLPF	9	100.00% (11/11)
	EFVFKNIDGYFKIYS	15	27.27% (3/11)
	QPYRVVVLSFELLHA	15	54.55% (6/11)
	KTSVDCTMY	9	72.73% (8/11)
	MTKTSVDCTMYICGD	15	27.27% (3/11)

### Vaccine Design

The finalized epitopes in Table 1 were examined for their interactive ability with one another using HADDOCK. All possible combinations of epitopes along with a flexible linker GGGGS between them were explored. For vaccine 1, the E1S1–E4S1 combination had the highest haddock refinement score.

The binding affinity of the E1S1–E4 S1 combination with the other two epitopes was determined to find the best combination of three epitopes. E1 S1 –E4 S1 –E3 S1 was thus formed. Finally, the vaccine construct obtained after combination analysis was E1 S1–E4 S1–E3 S1–E2 S1 (Table 3). Similarly, for vaccine 2, E1- S1 –E4 S1 was the first combination, and it was followed by E1S1 –E4 S1–E2 S2 and E1 S1–E4 S1–E2 S2 –E1 S2. Each probable combination lined up for putative vaccine design along with their corresponding HADDOCK scores is present in Table 3. Moreover, truncated Ov-Asp1 (IVVAVTGYNCPGGKLTALERKKIVGQNNKYRSDLINGKLKNRNGTYMPRGKNMLELTWDCKLESSAQRWANQCIFGHSPRQQREGVGENVYAYWSSVSVEGLKKTAGTDAGKSWWSKLPKLYENNPSNNMTWKVAGQGVLHFTQ) was attached to the *N* terminal of both the putative vaccines using another linker, EAAAK. The finalized vaccines together with the linkers and adjuvant were 212 amino acids long. Ov-ASP-1 reportedly has ability to activate antigen-processing cells (APCs) which define its good adjuvanticity for a number of vaccines and antigens (30). They are thus added in vaccine constructs to improve the efficacy of these new generation subunit vaccines.

**Table 3:** Potential multi-epitopic combinations with their corresponding HADDOCK refinement scores.

	**Best combinations**	**HADDOCK refinement score**
Vaccine 1	E1 S1–E4 S1	−79.1 +/– 2.3
	E1 S1 –E4 S1 –E3 S1	−113.2 +/– 2.5
	E1 S1–E4 S1–E3 S1–E2 S1	−123.7 +/– 1.3
Vaccine 2	E1 S1–E4 S1	−79.1 +/– 2.3
	E1 S1–E4 S1–E2 S2	−100.4 +/– 1.2
	E1 S1–E4 S1–E2 S2–E1 S2	−92.7 +/– 2.6
Vaccine 3	E4 S1- E5 S1	−128.4 +/– 1.7
	E4 S1- E5 S1- E2 S2	−96.6 +/– 0.4
	E4 S1- E5 S1- E2 S2- E1 S2	−96.6 +/– 0.4
	E4 S1- E5 S1- E2 S2- E1 S2- E1 S1	−77.2 +/– 1.3

**Figure 1:**
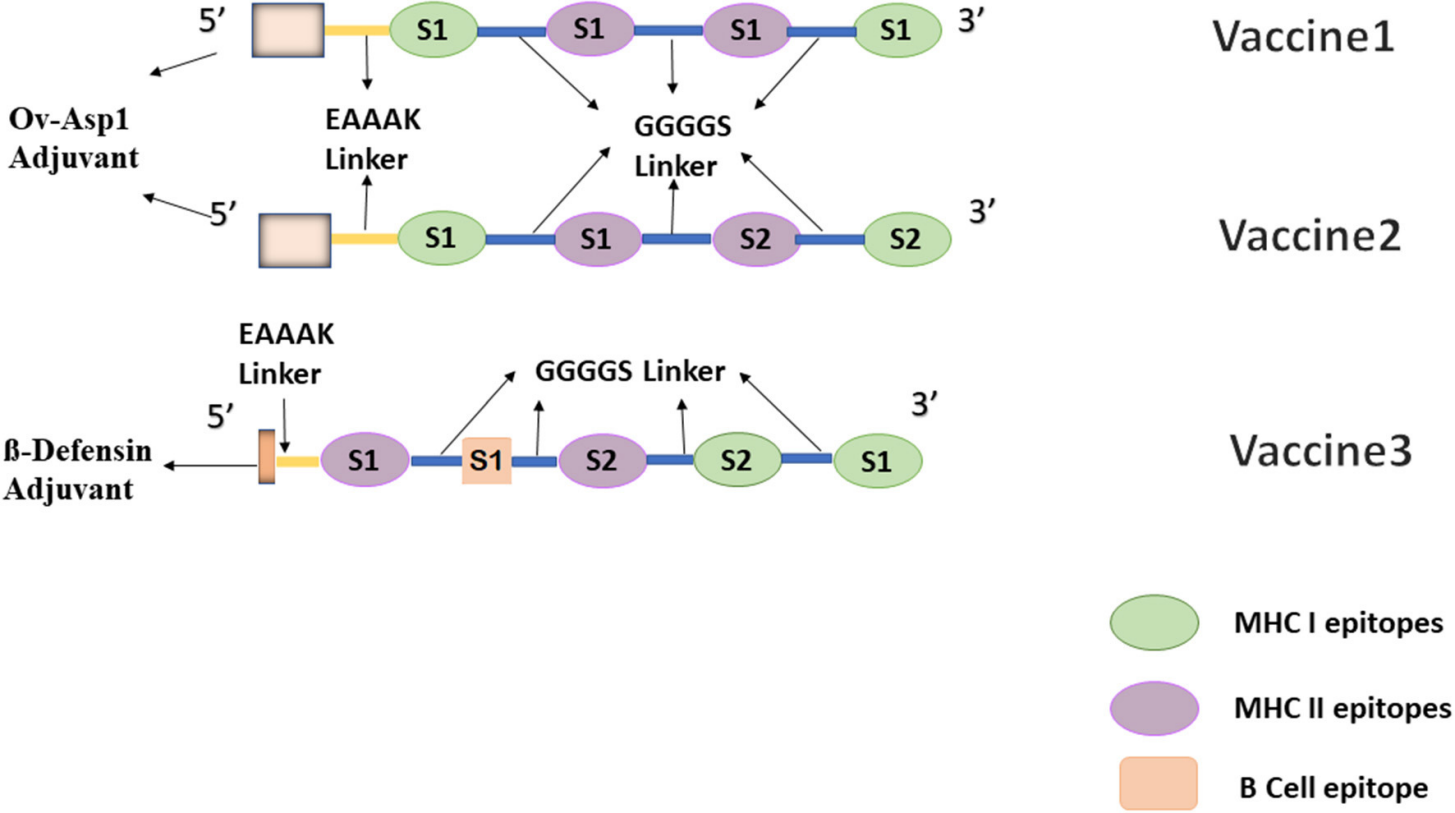
Graphical representation of designed multi-epitopic vaccine constructs.

In order to ensure both cell and humoral mediated responses, a potent B-cell epitope was added to vaccine 2 based on the best docking scores predicting the combination pattern of epitopes. Vaccine 3 was created with an order; E4 S1–E5 S1–E2 S2–E1 S2–E1 S1 and the corresponding docking score are enlisted in Table 3. For comparison purposes, another adjuvant beta-defensin (GIINTLQKYYCRVRGGRCAVLSCLPKEEQIGKCSTRGRKCCRRKK) was added to this combination. Beta defensin has previously been reported as a potent adjuvant when conjugated with MERS-CoV antigens (38). Vaccines containing defensins as adjuvants have been shown, both *in vivo* and *in vitro*, to activate the primary innate antiviral immune response and mediate other immunomodulatory activities against a number of viruses, including coronaviruses (38, 39). Vaccine 3, after addition of this adjuvant at the N terminal along with EAAAAK and GGGGS linkers, consisted of 143 residues. The final combination of epitopes of all three vaccine constructs have been shown in Figure 1.

### Evaluation of Physicochemical Parameters of the Chimeric Vaccine Construct

Various physiochemical properties were examined for both the constructs. The molecular weight of vaccine 1 is 23235.26 g/mol while the theoretical pI is 9.50, depicting the basic nature of the peptide construct. The instability index II showed that the construct is stable with a score of 24.79. The GRAVY (GRand AVerage of hydropathY) index was calculated to be −0.479, validating the hydrophilic nature of the construct that can form interactions with surrounding water molecules. The aliphatic index 67.12 illustrated that the construct is thermostable in nature.

Vaccine 2 has a molecular weight of 23013.07 g/mol, and its theoretical pI is 9.33. Hence, this construct was also found to be basic in nature. Likewise, instability analysis showed that the protein is stable with a score of 24.50. The GRAVY index testified the hydrophilic nature of this construct as well (−0.492). The thermostable nature of the construct was established by the value of aliphatic index, 62.97. The predicted values of antigenicity for both the vaccines were found to be 0.883591 and 0.946425, respectively. This ensured highly antigenic nature of the constructs. Similarly, the solubility upon overexpression was predicted to be 0.864955 and 0.951926. Furthermore, both vaccine constructs designed in this study were designated as non-allergenic by AllergenPro.

Vaccine 3 has a molecular weight of 15084.28 g/mol and its theoretical pI is 9.25. Therefore, this vaccine construct was also found to be basic in nature. The GRAVY index testified the hydrophilic nature of this construct as well (−0.253). The thermostable nature of the construct was established by the value of aliphatic index, 55.87. The predicted values of antigenicity for this particular the vaccine was 0.883570. This ensured highly antigenic nature of the construct. Similarly, the solubility upon overexpression was predicted to be 0.806206. Furthermore, like both the previous vaccine constructs designed in this study, this vaccine was also found to be non-allergenic by AllergenPro.

### Secondary and Tertiary Structure Prediction

The secondary structure of vaccine 1 includes six helices, 35 beta turns, seven gamma turns, and nine helix-helix interactions. The secondary structure of vaccine 2 has eight helices, 22 beta turns, 12 gamma turns, and nine helix–helix interactions. For vaccine 3, secondary structure consisted of two beta strand, one hairpin, one sheet, four helices, 23 beta turns, 23 gamma turns, and one helix–helix interaction. Helix–helix interaction presents facts about different pairs of helices, interacting with each other with the vicinity of the protein structure, whereas beta turns depict four consecutive residues. These four residues are represented by i, i + 1, i + 2, and i + 3. This is possible when the measured distance between the alpha Carbon atom of the first residue (i) and alpha carbon atom of the fourth residue (i + 3) is <7 Å plus the two residues between them are not helical. A gamma turn comprises of three residues i, i + 1, and i + 2. This is possible when a hydrogen bond is present between the two residues (i.e., i and i + 2). Moreover, the phi angle and the psi angle of the second residue i.e., i + 1 lies within a range of 40 degrees in one of the next two cases: (1) classic [phi i + 1(75), psi i + 1(−64)] or (2) inverse [phi i + 1(−79), psi i + 1(−69)].

**Figure 2:**
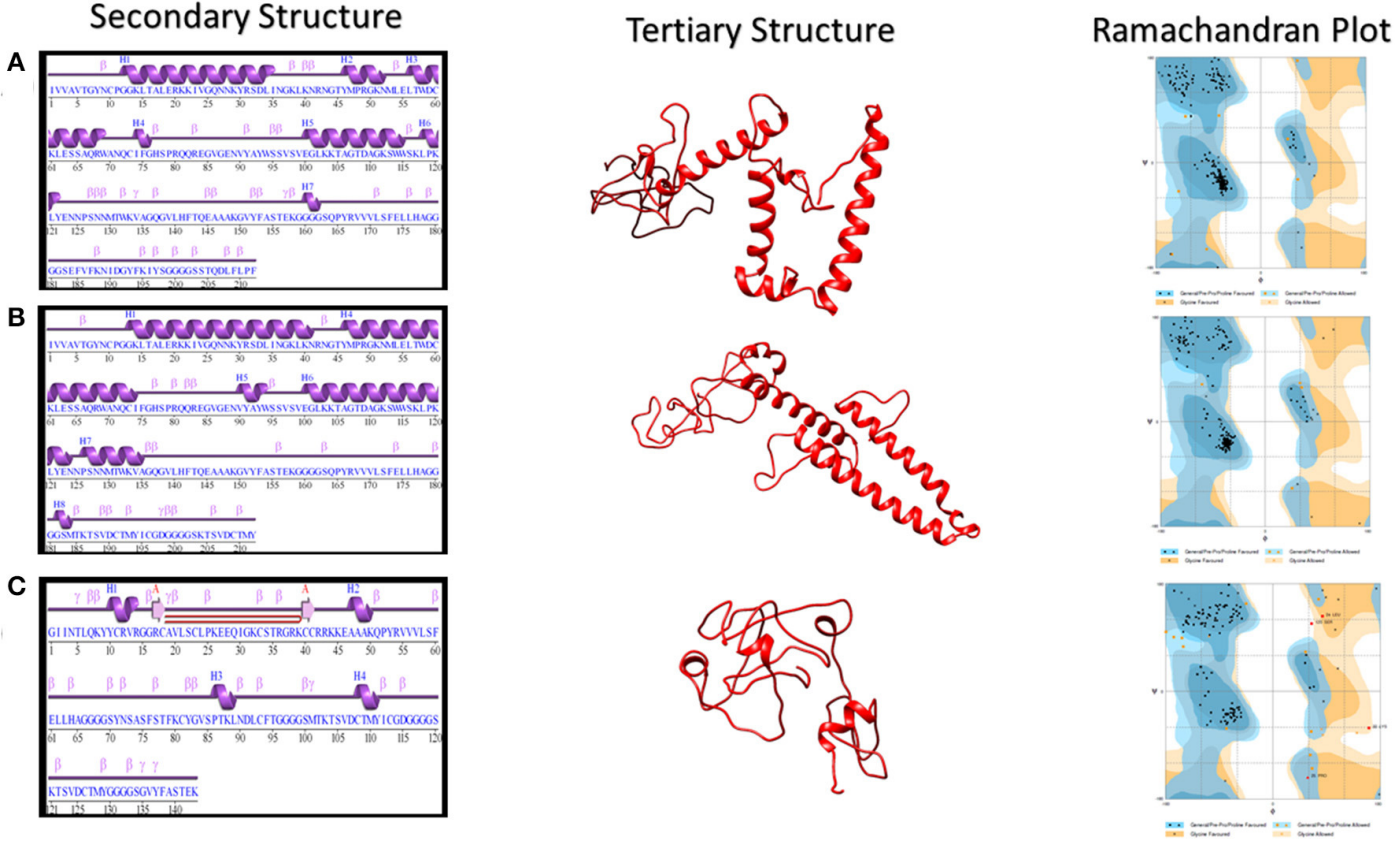
Secondary and Tertiary structures of proposed vaccine constructs. **(A)** Secondary and Tertiary structures of vaccine 1 along with its Ramachandran Plot analysis, which showed 97.1% residues in the favored region and 2.9% in the allowed region. **(B)** Secondary and Tertiary structures of vaccine 2 along with its Ramachandran Plot analysis, which showed 98.1% residues in the favored region while 1.9% in the allowed region. **(C)** Secondary and Tertiary structures of vaccine 3 along with its Ramachandran Plot analysis, which showed 86.5% residues in the favored region.

The 3Dpro tool, which works on the basis of ab initio method for predicting tertiary structure, was used to predict three dimensional structures of proposed vaccine constructs. This strategy was adopted due to the lack of fine homolog proteins that could be exploited for homology modeling. The obtained models were then refined via several structure perturbations and subsequent structure relaxations using GlaxyRefine server. The obtained best models are shown in Figure 2. The ERRAT score for 3D models of three vaccines were calculated as 74.1379, 67.5676, and 74.2574, respectively. While Ramachandran plot analysis showed 97.1% residues in favored region for vaccine 1, 98.1% residues in the favored region for vaccine 2 and 86.5% for vaccine 3 (Figure 2). These analyses authenticated the reliability and stability of the predicted structures.

Energy minimization by a YASARA server was performed. For vaccine 1, the YASARA force field was applied to 2,032 atoms. A total of 5,282 water molecules were found. The initial energy was −68794.3kJ/mol (*Z* score −1.90), which was minimized to −97974.1 kJ/mol (−1.93). For vaccine 2, the YASARA force field was applied to 2,006 atoms while the water molecules were 5,208. Initial energy was −66687.0 kJ/mol (*Z* score −2.08); however, the final energy was 101214.7 kJ/mol (*Z* score −1.47). For vaccine 3, the YASARA force field was applied to 2,093 atoms while the water molecules were 4,185. Initial energy was −53609.9 kJ/mol (*Z* score −3.35); however, the final energy was −60374.6 kJ/mol (*Z* score −3.16).

### Interaction of Predicted Vaccines With Potential Receptors

**Figure 3:**
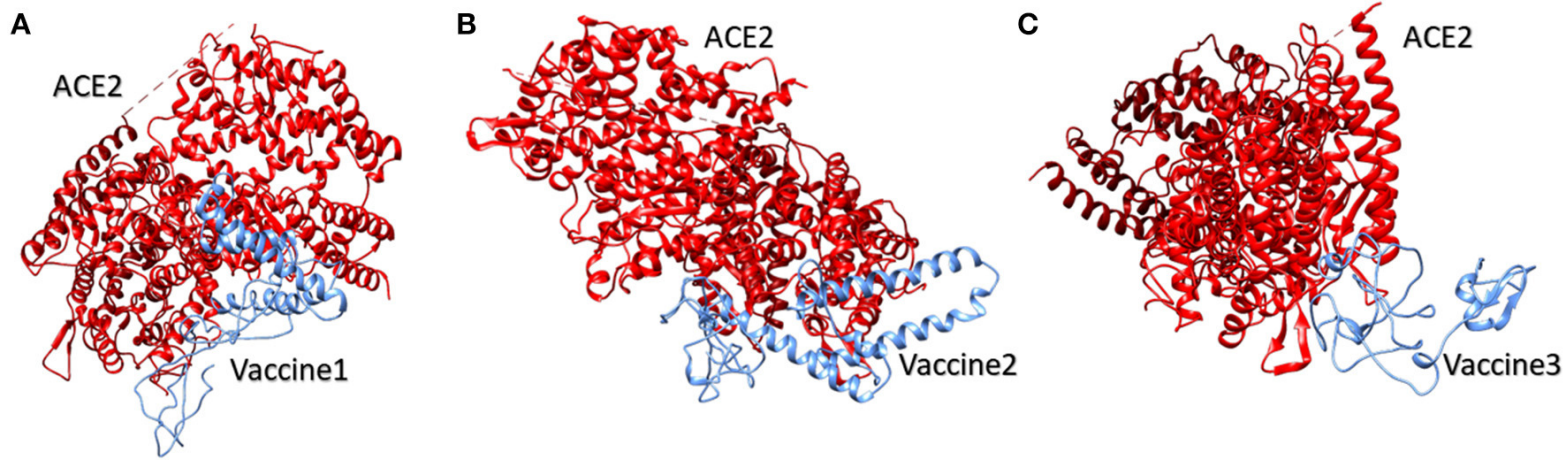
Human ACE2 protein complex with proposed multi-epitopic COVID-19 vaccines. **(A)** Designed vaccine 1 (blue) interacting with receptor ACE2 (red). **(B)** Designed vaccine 2 (blue) interacting with receptor ACE2 (red). **(C)** Designed vaccine 3 (blue) interacting with receptor ACE2 (red). These interactions have been predicted via docking results obtained by HADDOCK.

SARS-CoV spike protein has been studied previously for its exceptional binding affinity with human ACE-2. It should be noted that, structurally, SARS-CoV-2 and SARS-CoV spike proteins are highly homologous in nature, sharing 76.5% identical amino acids. Atomic level studies between SARS-CoV and ACE-2 show promising interactions between the two, and therefore, owing to the structural and sequence similarity, it is anticipated that an ACE-2 blocker might be handy in curbing SAR-CoV-2 (40). For vaccine 1 and the ACE-2 receptor, therefore, docking was carried out. A HADDOCK server clustered 36 probable structures into seven different clusters, which represented a total of 18.0 % of the water-refined models. The top-scoring cluster had a score of 39.8 +/– 29.1 and a *Z* score of −1.6. Similarly, for vaccine 2 and the ACE-2 receptor, HADDOCK clustered 18 structures in three clusters, which represented 9.0% of the water-refined models. The top-scoring cluster had a value of 0.3 +/– 9.8 and a *Z* score of −1.3. Likewise, for vaccine 3, HADDOCK clustered 22 structures into five clusters, which depicted 11% of the water refined models generated by HADDOCK. Here, the best cluster had a score of 147.5 +/– 15.0 and a *Z* score of −1.2 (Figure 3).

TLR2 and TLR4 are well-studied Toll-Like Receptors that identify both structural and non-structural proteins of the virus and subsequent cytokine production and inflammation. They are present on the surface of cells and are triggered by viral glycoproteins. TLR agonists have the potential to initiate an immune response and actively participate in viral clearance (41). The prioritized vaccine constructs were therefore also explored for their interaction with Toll-like receptors TLR2 and TLR4. Vaccine 1 and TLR2 interaction revealed 40 structures in a total of six clusters that represented 20.0% of the water-refined models. The model with the highest score, −4.2 +/– 20.8 had a Z value of −1.2. Likewise, for vaccine 2 and TLR2, HADDOCK clustered 80 structures in 12 clusters, which represented 40.0% of the water-refined models. Here, the highest-scoring model had a score of −23.7 +/– 12.1 with a *Z*-value of −1.3. For vaccine 3 and TLR2, HADDOCK clustered 136 structures in 10 clusters, which represented 68.0% of the water-refined models. The highest scoring model had a score of −16.7 +/– 14.0 with a *Z*-value of −1.8.

Moreover, HADDOCK clustered 157 structures in 13 clusters to determine vaccine 1 and TLR4 interaction, which represented 78.5% of the water-refined models. The top-scoring model had a score of 37.9 +/– 7.8 and a *Z*-value of −2.2, whereas the interaction of vaccine 2 and TLR4 is determined by 47 structures in nine cluster(s), which represents 23.5% of the water-refined models. The top-scoring model had a score of −16.8 +/– 23.4 (*Z*-value −1.6). Similarly, HADDOCK clustered 93 structures in eight clusters to determine vaccine 3 and TLR4 interaction, which represented 46.5 % of the water-refined models. The top-scoring model had a score of 23.3 +/– 5.7 and a *Z*-value of −1.3.

**Figure 4:**
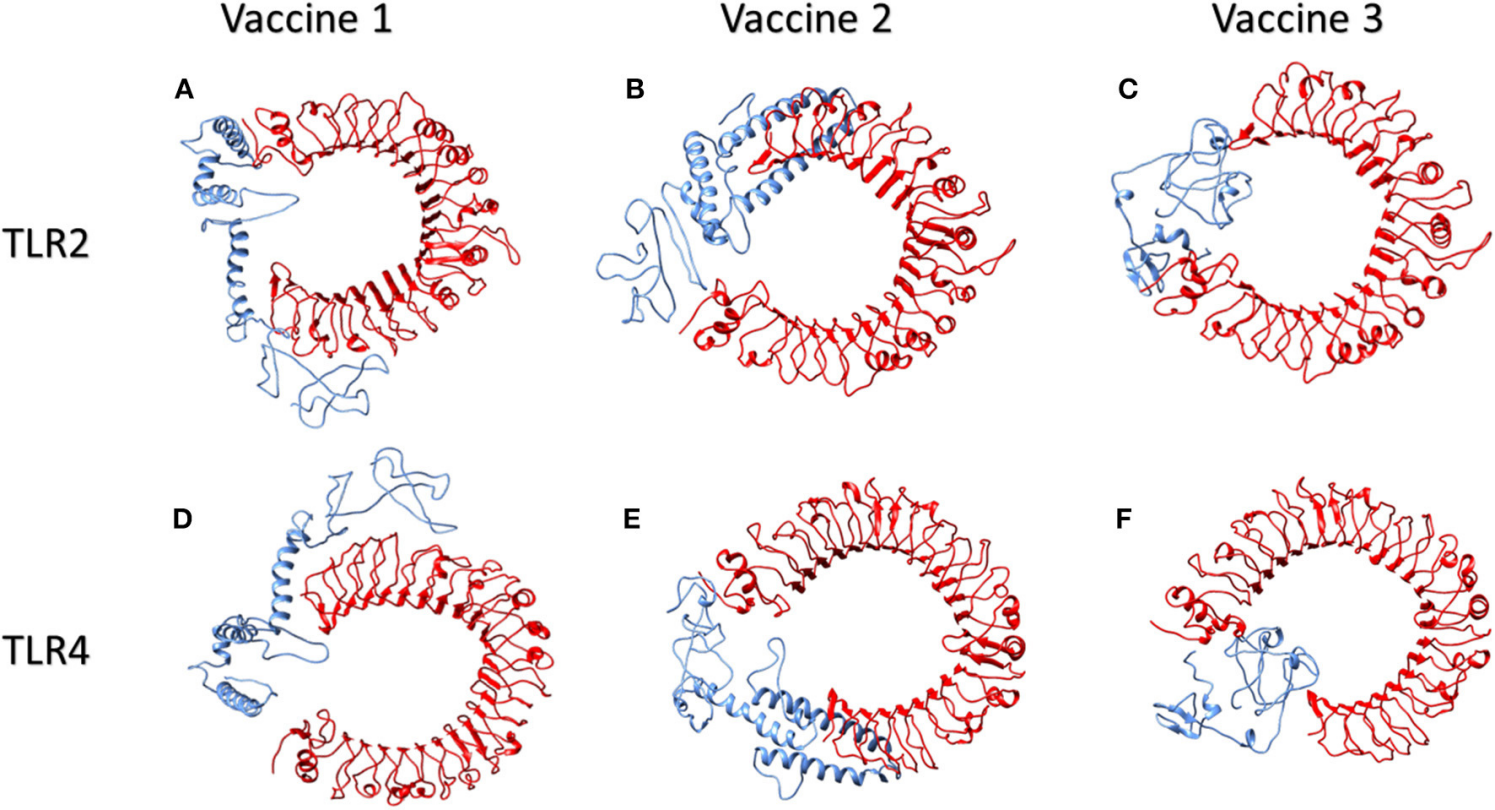
Human TLR2 and TLR4 proteins in complex with proposed multi-epitopic COVID-19 vaccines. **(A)** TLR2 (red) complex with proposed vaccine 1 (blue). **(B)** TLR2 (red) complex with proposed vaccine 2 (blue). **(C)** TLR2 (red) complex with proposed vaccine 3 (blue). **(D)** TLR4 (red) complex with proposed vaccine 1 (blue). **(E)** TLR4 (red) complex with proposed vaccine 2 (blue). **(F)** TLR4 (red) complex with proposed vaccine 3 (blue).

Models from top clusters were refined using HADDOCK refinement interface. This server was used to cluster 20 structures, obtained via HADDOCK, into one cluster. This final cluster symbolized 100% of water-refined models that were generated by HADDOCK. The statistics observed in interactions of vaccine 1, vaccine 2, and vaccine 3 from their refined clusters can be seen in Table 4, and complexes are shown in Figure 4.

**Table 4:** Table presenting statistics of interaction of all three vaccine constructs with ACE2, TLR2, TLR4, and BCR.

**Parameters**	**Vaccine 1**			**Vaccine 2**			**Vaccine 3**			
	**ACE2**	**TLR2**	**TLR4**	**ACE2**	**TLR2**	**TLR4**	**ACE2**	**TLR2**	**TLR4**	**BCR**
HADDOCK score	−256.0 +/– 4.0	−207.5 +/– 2.4	−132.7 +/– 1.5	−159.2 +/– 2.3	−160.9 +/– 3.3	−209.0 +/– 4.5	−114.9 +/– 2.0	−111.7 +/– 6.7	−163.6 +/– 0.7	−172.7 +/– 1.6
Cluster size	20	20	20	20	20	20	20	20	20	20
RMSD from the overall lowest-energy structure	0.3 +/– 0.2	0.3 +/– 0.2	0.3 +/– 0.2	0.3 +/– 0.2	0.3 +/– 0.2	0.3 +/– 0.2	0.3 +/– 0.2	0.3 +/– 0.2	0.3 +/– 0.2	0.3 +/– 0.2
Van der Waals energy	−179.7 +/– 5.6	−134.2 +/– 5.7	−86.9 +/– 3.1	−118.5 +/– 3.2	−114.2 +/– 4.1	−129.3 +/– 4.5	−92.3 +/– 3.2	−79.2 +/– 4.5	−88.2 +/– 4.4	−91.0 +/– 2.5
Electrostatic energy	−346.1 +/– 31.9	−346.2 +/– 9.9	−106.1 +/– 11.4	−296.9 +/– 7.0	−324.6 +/– 17.7	−474.5 +/– 7.7	−206.5 +/– 11.7	−358.5 +/– 31.4	−452.3 +/– 20.2	−182.0 +/– 16.1
Desolvation energy	−7.0 +/– 3.2	−4.1 +/– 3.1	−24.6 +/– 2.8	18.7 +/– 2.2	18.2 +/– 3.7	15.3 +/– 6.9	18.7 +/– 2.8	39.2 +/– 7.9	15+/−4.2	−45.3 +/– 2.5
Restraints violation energy	0.2 +/– 0.08	0.0 +/– 0.00	0.0 +/– 0.00	0.3 +/– 0.20	0.0 +/– 0.00	0.0 +/– 0.00	0.2 +/– 0.04	0.0 +/– 0.00	0.0 +/– 0.00	0.0 +/– 0.00
Buried Surface Area	4957.9 +/– 37.1	3649.0 +/– 44.9	2323.3 +/– 31.9	3763.3 +/– 92.5	2977.2 +/– 57.0	3525.8 +/– 51.9	2572.8 +/– 16.3	2356.9 +/– 45.1	2764.0 +/– 31.3	2405.1 +/– 21.9

*Here, a lower HADDOCK score indicates the higher strength of interaction between the proteins. Z-score of all docking complexes came out to be 0*.

The PDBsum analysis of vaccine 1 with ACE2 showed 18 hydrogen bonds and one salt bridge. Additionally, 42 interface residues of vaccine 1, representing an interface area of 2,502 (A^2^), were found while the corresponding ACE2 had 45 interface residues covering an area of 2,319 (A^2^). For vaccine 2 and ACE2, there were two salt bridges and seven hydrogen bonds predicted by PDBsum. Additionally, 28 and 38 residues from vaccine 2 and ACE2 interacted with each other covering an area of 1,997 and 1,832, respectively. Likewise, for vaccine 3 there was one salt bridge and 13 hydrogen bonds predicted by PDBsum. Additionally, 27 and 22 residues from vaccine 3 and ACE2 interacted with each other, covering an area of 1,228 and 1,271, respectively.

An interaction analysis of vaccine 1 with the TLR2 interacting complex via PDBsum exhibited 19 hydrogen bonds and one salt bridge. Furthermore, 35 interface residues of vaccine 1, representing an interface area of 1,795 (A^2^), were found while a corresponding TLR2 had 36 interface residues encompassing an area of 1,880 (A^2^). For vaccine 2 and TLR2, there were two salt bridges and 14 hydrogen bonds predicted by PDBsum. Additionally, 23 and 25 residues from vaccine 2 and TLR2 interacted with each other, covering an area of 1,362 and 1,443, respectively. Lastly, for vaccine 3 and TLR2 PDBsum, 17 hydrogen bonds and five salt bridges were found. Furthermore, 25 interface residues of vaccine 3, representing an interface area of 1,104 (A^2^), were found while corresponding a TLR2 had 21 interface residues, encompassing an area of 1,194 (A^2^).

Similarly, the interaction of vaccine 1 with TLR4 exhibited eight hydrogen bonds and 18 interface residues of vaccine 1, representing an interface area of 1,171 (A^2^) while a corresponding TLR4 had 19 interface residues, encompassing an area of 1,146 (A^2^). For vaccine 2 and TLR4, there were three salt bridges and 20 hydrogen bonds predicted by PDBsum. Additionally, 33 and 34 residues from vaccine 2 and TLR4 interacted with each other, covering an area of 1,763 and 1,745, respectively. In case of vaccine 3 and TLR4, 15 hydrogen bonds and three salt bridges were found, while 34 interface residues of vaccine 3 represented an interface area of 1,397 (A^2^) and a corresponding TLR4 had 27 interface residues, encompassing an area of 1,396 (A^2^).

For the interaction analysis of vaccine 3 and BCR (CD79), the HADDOCK server clustered 140 probable structures into 13 different clusters, which represented a total of 70% of the water-refined models. The top scoring cluster had a score of −43.6 +/– 16.0 and a *Z* score of −1.7. Models from top clusters were refined using HADDOCK refinement interface. This server was used to cluster 20 structures, obtained via HADDOCK, into one cluster. This final cluster symbolized 100% of water-refined models that were generated by HADDOCK. The statistics observed in interactions of vaccine 3 and BCR from its particular refined clusters can be seen in Table 4. Pdbsum analysis showed that 26 and 18 residues from vaccine 3 and BCR interacted with each other covering an interface area (A2) of 1,181 and 1,205, respectively. They formed one salt bridge and 10 hydrogen bonds.

### Interaction of Proposed Vaccines With HLA Alleles

For interaction analysis of vaccine 1 and HLA A allele, the HADDOCK server clustered 118 probable structures into 17 different clusters, which represented a total of 59.0 % of the water-refined models. The top scoring cluster had a score of −26.5 +/– 2.7 and a *Z* score of −2.5. Similarly, for vaccine 2 and the HLA A allele, HADDOCK clustered 97 structures in 17 clusters, which represented 48.5 % of the water-refined models. The top scoring cluster had a value −57.5 +/– 12.8 and a *Z* score of −2.3. Likewise, for vaccine 3 HADDOCK clustered 187 structures into three clusters, which depicted 93.5% of the water refined models generated by HADDOCK. Here the best cluster had a score of −34.7 +/– 1.9 and a *Z* score of −1.1. For vaccine 1 and HLA B allele, 115 probable structures were clustered by HADDOCK into 15 different clusters, which represented a total of 57.5 % of the water-refined models. The top scoring cluster had a score of −57.5 +/– 12.8 and a *Z* score of −2.3. Similarly, for vaccine 2 and the HLA B allele, HADDOCK clustered 84 structures into nine clusters, which represented 42% of the water-refined models. The top scoring cluster had score of −18.7 +/– 8.7 and *Z* score of −1.6. Likewise, for vaccine 3 HADDOCK clustered 168 structures into 10 clusters, which depicted 84% of the water refined models were generated. Here, the best cluster had a score of −41.2 +/– 18.7 and a *Z* score of −2.1.

Furthermore, for vaccine 1 and the HLA DRB1 allele docking, the HADDOCK server clustered 67 probable structures into 10 different clusters, which represented a total of 33.5 % of the water-refined models. The top-scoring cluster had a score of −27.8 +/– 6.0 and a *Z* score of −2.3. Similarly, for vaccine 2 and HLA DRB1 allele, HADDOCK clustered 64 structures in 11 clusters, which represented 32% of the water-refined models. The top-scoring cluster had score of −24.8 +/– 25.6 and *Z* score of −1.7. Likewise, for vaccine 3, HADDOCK clustered 93 structures into 13 clusters, which depicted 46.5% of the water refined models generated by HADDOCK. Here the best cluster had a score of −37.1 +/– 11.8 and a *Z* score of −1.5. Models from top clusters were refined using HADDOCK refinement interface. This server was used to cluster 20 structures, obtained via HADDOCK, into one cluster. This final cluster symbolized 100% of water-refined models that were generated by HADDOCK. The statistics observed in interactions of vaccine 1, vaccine 2, and vaccine 3 from their particular refined clusters can be seen in Table S4.

### Population Coverage

Epitope population coverage was checked by IEDB population coverage tool. Resultantly, all epitopes had a combined Class I and Class two average coverage score of 94%. This step was performed by using the entire world population datasets and the MHC restricted alleles used in this case were (A^*^01:01, A^*^02:01, A^*^03:01, A^*^24:02, A^*^26:01, B^*^07:02, B^*^08:01, B^*^27:05, B^*^39:01, B^*^40:01, B^*^58:01, B^*^15:01, HLA-DRB1^*^03:01, HLA-DRB1^*^07:01, and HLA-DRB1^*^15:01).

## Discussion

Coronavirus can reportedly spread from person to person via droplet transmission. However, there is currently no available FDA-approved vaccine against COVID-19 (42, 43). A vaccination regime, if successfully developed against COVID-19, has the ability to improve global human health statistics. The advent of immuno-informatics approaches has revolutionized the area of vaccine development. Antibody response as well as cell mediated immunity can be established by using proper protein antigens (44). Notably, the natural infections elicit a minimal immune response that can be enhanced by developing epitope-based vaccines. Therefore, rational selections are done to separate the constituents required for the desired immune response. Efforts to identify suitable T-cell epitopes as well as the design of effective strategies in order to deliver those epitopes are under consideration. The benefits of epitope-based vaccine construction includes improved safety levels, time saving, and, additionally it can provide the opportunity to specifically attach/engineer combinations of epitopes for augmented potency. This also facilitates to emphasize the required immune responses on antigenic/ conserved epitopes (45).

Spike proteins of coronaviruses are responsible for selection and entry into the target cells. Any therapeutic approach to target the spike protein can prove to be fruitful to curb the deadly pathogen. Moreover, it has been reported that like SARS-CoV, SARS-CoV-2 uses the ACE2 human receptor to bind and enter the cells (12). Peptides that potentially interact with the functional domain of the coronavirus Spike protein, can be designated as viral entry inhibitors. In our study, the chosen CD4+ and CD8+ T cell epitopes are predicted to be antigenic and immunogenic, and they can thus play a vital role in viral clearance mechanisms. To further validate the authenticity of our proposed vaccines, more detailed docking analysis and experimentation has to be performed. Nevertheless, it might take months to years to actually derive a vaccine against COVID-19, we believe that our contribution in this case might be a useful to initiate the process.

For vaccine 1, four epitopes from the S1 domain were picked. The S1 domain, which comprises of amino acids from 14 to 685, is further divided into the N-terminal domain and receptor-binding domain. Analysis showed that three of our chosen epitopes lied in the N-terminal domain of the S1 protein while one “^506^QPYRVVVLSFELLHA^520^” was a part of receptor-binding domain (319-541). Viral infections are prompted by the interaction of the spike-protein with the receptor, present on the surface of the target cell. This process is mediated by the receptor binding portion of the S1 domain. Hence, it plays a significant role in the attachment, and subsequent fusion and entry of the virus into the host cell. Hence this particular portion can be targeted for designing antiviral agents (46).

Vaccine 2 is comprised of a combination of strong and weak epitopes. It had two epitopes (MHC-I and MHC-II) that were found to be the best epitopes for S1 domain. Regardless of the fact that ^91^EFVFKNIDGYFKIYS^205^ had a higher antigenicity score compared to ^506^QPYRVVVLSFELLHA^520^ (1.0339 and 0.9109), the latter was used in vaccine construction due to its presence in receptor binding domain. Additionally, another experimental strategy was applied; comparatively weak epitopes from S2 were selected and their binding affinity was checked. Docking with TLRs and ACE2 showed that they bind effectively; from ^731^MTKTSVDCTMYICGD^745^, Thr^192^, Val^197^, Lys^186^, Thr^187^, and Ser^186^ bound to TLR4; Lys^186^ had affinity for ACE2 receptor. The other S2 epitope ^733^KTSVDCTMY^741^ completely overlapped with ^731^MTKTSVDCTMYICGD^745^.

Vaccine 3 is a modified form of vaccine 2 with an additional 25mer B-cell epitope ^369^YNSASFSTFKCYGVSPTKLNDLCFT^393^ integrated. The whole idea to include B-cell epitopes along with was to ensure both cellular and humoral defense responses (47). B-cell epitopes are precisely amino acids clusters present at the cell surfaces that are identified by certain antibodies or B-cell receptors, that in turn elicit cellular or hormonal immune response (48). Antibodies released by B-cells can neutralize toxins and thus label them for destruction (49, 50). In this case, in addition to considerable interactions with TLRs and HLA superfamily alleles, notable interactions were observed between Cys^93^ and Phe^94^ from B cell epitope and Arg^8^ and Glu^96^ from BCR, respectively.

Designed vaccines have been tested against different receptors to identify their potential to induce immune response within the host. Results revealed that proposed vaccines are likely to be presented by MHC-I and MHC-II, as that was the prime objective of this study. Also, they may interact with human TLR2 and TLR4 to induce innate immune response, as these receptors have been revealed to play a key role in the induction of immune responses (51). Moreover, the Spike protein of SARS-COV has been reported to play a significant role in the induction of neutralizing-antibodies and T-cell responses as well as protective immunity during the infection (52). Therefore, keeping in view the importance of spike proteins in immunity, we applied this predictive framework to identify potential vaccine candidates in spike protein of SARS-CoV-2 against its potential host receptor ACE2 as well as against TLR4 and TLR2. Recent studies have strongly suggested that COVID-19 uses angiotensin-converting enzyme 2 (ACE2) as its potential receptor. Several critical residues in COVID-19 receptor binding motif (RBM) of S1 domain particularly Gln493 provide favorable interactions with human ACE2 (53). Thus, it has been proposed in several studies that spike-protein-based vaccines can be potential therapeutic targets against SARS-CoV-2, as they may block the viral interaction with ACE2 and may thus prevent the downregulation of ACE2 and ultimately the pulmonary vascular permeability (54). Vaccines designed in this study may also interact with ACE2 resulting interrupted interaction of the receptor with the viral spike protein and thus can be a potential therapeutic target against COVID-19. The overall effect of all these interactions within the host is still unknown and requires further experimental studies for their clear role in the immune regulation and virus clearance.

## Conclusions

Concisely, we have combined several immuno-informatics tools to propose a set of potentially antigenic and immunogenic peptide epitopes that can facilitate vaccine design. The predicted vaccine constructs consist of distant epitopes. The authenticity of these constructs must be validated via further experimentation. However, further experimental authentication is required to verify this study. We anticipate promising outcomes from the predicted peptide epitopes to curb the deadly COVID-19 pandemic.

## Data Availability Statement

All datasets presented in this study are included in the article/Supplementary Material.

## Author Contributions

AN, FS, and FA conceptualized the study, validated the study, and wrote the original draft. FS performed the data curation and developed the software. AN and FS performed the formal analysis, methodology, and visualized the study. TB, AA, and AM performed the funding acquisition. AN performed the investigation and supervised the study. AN and AM performed the project administration. AN, TB, FA, AA, and AM wrote, reviewed, and edited the manuscript. All authors contributed to the article and approved the submitted version.

## Conflict of Interest

The authors declare that the research was conducted in the absence of any commercial or financial relationships that could be construed as a potential conflict of interest.
