# Supplementary material for: Designing Multi-Epitope Vaccines to Combat Emerging Coronavirus Disease 2019 (COVID-19) by Employing Immuno-Informatics Approach
Source: Front Immunol. 2020 Jul 10;11:1663. doi: 10.3389/fimmu.2020.01663 (PMC7365865; doi:10.3389/fimmu.2020.01663)
Supplement: Supplementary file 1 [file Data_Sheet_1.docx]

Supplementary Material

# Supplementary Tables

## Supplementary Table S1: Predicted T cell epitopes (MHC I ) of the COVID-19 surface protein sequence, their location (aa) , percentile rank along with antigenicity and immunogenicity scores.

| S. No. | Peptide location (start) | Peptide location (end) | Peptide | IEDB consensus percentile rank | Strongest binding Allele | Antigenicity score | Immunogenicity score |
| --- | --- | --- | --- | --- | --- | --- | --- |
| 1 | 865 | 873 | LTDEMIAQY | 0.11 | A*01:01 | 0.1043 | 0.02757 |
| 2 | 258 | 266 | WTAGAAAYY | 0.17 | A*01:01 | 0.6306 | 0.15259 |
| 3 | 269 | 277 | YLQPRTFLL | 0.3 | A*02:01 | 0.4532 | 0.1305 |
| 4 | 1220 | 1228 | FIAGLIAIV | 0.4 | A*02:01 | 0.3162 | 0.27206 |
| 5 | 454 | 462 | RLFRKSNLK | 0.1 | A*03:01 | 0.2829 | -0.28759 |
| 6 | 89 | 97 | GVYFASTEK | 0.2 | A*03:01 | 0.7112 | 0.09023 |
| 7 | 1208 | 1216 | QYIKWPWYI | 0.11 | A*24:02 | 1.4177 | 0.21624 |
| 8 | 635 | 643 | VYSTGSNVF | 0.17 | A*24:02 | -0.3099 | -0.11871 |
| 9 | 192 | 200 | FVFKNIDGY | 0.11 | A*26:01 | -0.1304 | -0.0215 |
| 10 | 258 | 266 | WTAGAAAYY | 0.11 | A*26:01 | 0.6306 | 0.15259 |
| 11 | 680 | 688 | SPRRARSVA | 0.1 | B*07:02 | 0.7729 | 0.0402 |
| 12 | 24 | 32 | LPPAYTNSF | 0.6 | B*07:02 | 0.3775 | -0.03341 |
| 13 | 342 | 350 | FNATRFASV | 0.2 | B*08:01 | 0.5609 | 0.14872 |
| 14 | 869 | 877 | MIAQYTSAL | 0.3 | B*08:01 | 0.1114 | -0.18768 |
| 15 | 453 | 461 | YRLFRKSNL | 0.4 | B*27:05 | 0.0522 | -0.1818 |
| 16 | 236 | 244 | TRFQTLLAL | 0.6 | B*27:05 | 0.3406 | -0.0377 |
| 17 | 236 | 244 | TRFQTLLAL | 0.11 | B*39:01 | 0.3406 | -0.0377 |
| 18 | 764 | 772 | NRALTGIAV | 0.23 | B*39:01 | 0.5302 | 0.20642 |
| 19 | 1016 | 1024 | AEIRASANL | 0.12 | B*40:01 | 0.7082 | 0.00689 |
| 20 | 168 | 176 | FEYVSQPFL | 0.28 | B*40:01 | 0.6324 | -0.17076 |
| 21 | 625 | 633 | HADQLTPTW | 0.3 | B*58:01 | 0.3807 | -0.0703 |
| 22 | 712 | 720 | IAIPTNFTI | 0.3 | B*58:01 | 0.7052 | 0.18523 |
| 23 | 50 | 58 | STQDLFLPF | 0.3 | B*15:01 | 0.6619 | 0.06828 |
| 24 | 1054 | 1062 | QSAPHGVVF | 0.4 | B*15:01 | 0.2234 | 0.1239 |

## Supplementary Table S2: Predicted T cell epitopes (MHC class II) of the surface protein of COVID-19 surface glycoprotein sequence, their location, median consensus percentile rank along with antigenicity scores.

| S.No | Peptide location (start) | Peptide location (end) | Peptide | Antigenicity score |
| --- | --- | --- | --- | --- |
| 1 | 971 | 985 | GAISSVLNDILSRLD | -0.0775 . |
| 2 | 236 | 250 | TRFQTLLALHRSYLT | 0.3262 . |
| 3 | 461 | 475 | LKPFERDISTEIYQA | -0.0873 . |
| 4 | 896 | 910 | IPFAMQMAYRFNGIG | 1.2828 . |
| 5 | 821 | 835 | LLFNKVTLADAGFIK | 0.6327 . |
| 6 | 231 | 245 | IGINITRFQTLLALH | 0.8391 |
| 7 | 451 | 465 | YLYRLFRKSNLKPFE | 0.0814 . |
| 8 | 1216 | 1230 | IWLGFIAGLIAIVMV | 0.6150 |
| 9 | 506 | 520 | QPYRVVVLSFELLHA | 0.9109 . |
| 10 | 1061 | 1075 | VFLHVTYVPAQEKNF | 1.0339 . |
| 11 | 306 | 320 | FTVEKGIYQTSNFRV | 0.2396 |
| 12 | 911 | 925 | VTQNVLYENQKLIAN | 0.0783 . |
| 13 | 156 | 170 | EFRVYSSANNCTFEY | 0.0802 |
| 14 | 51 | 65 | TQDLFLPFFSNVTWF | 0.3569 |
| 15 | 116 | 130 | SLLIVNNATNVVIKV | 0.4707 . |
| 16 | 1016 | 1030 | AEIRASANLAATKMS | 0.8255 |
| 17 | 191 | 205 | EFVFKNIDGYFKIYS | 1.0339 |
| 18 | 316 | 330 | SNFRVQPTESIVRFP | 0.2635 |
| 19 | 341 | 355 | VFNATRFASVYAWNR | 0.0745 |
| 20 | 56 | 70 | LPFFSNVTWFHAIHV | 0.6624 |

## Supplementary Table S3: Conservation pattern of COVID-19 prioritized epitopes among all available 43 coronavirus genomes.

| **Description** | **Accession** | **GVYFASTEK** | **STQDLFLPF** | **EFVFKNIDGYFKIYS** | **KTSVDCTMY** | **MTKTSVDCTMYICGD** | **QPYRVVVLSFELLHA** |
| --- | --- | --- | --- | --- | --- | --- | --- |
| Chain A, Spike glycoprotein [Severe acute respiratory syndrome coronavirus 2] | 6VSB_A | ✔ | ✔ | ✔ | ✔ | ✔ | ✔ |
| surface glycoprotein [Severe acute respiratory syndrome coronavirus 2] | YP_009724390.1 | ✔ | ✔ | ✔ | ✔ | ✔ | ✔ |
| surface glycoprotein [Severe acute respiratory syndrome coronavirus 2] | QIA20044.1 | ✔ | ✔ | ✔ | ✔ | ✔ | ✔ |
| surface glycoprotein [Severe acute respiratory syndrome coronavirus 2] | QHW06059.1 | ✔ | ✔ | ✔ | ✔ | ✔ | ✔ |
| surface glycoprotein [Severe acute respiratory syndrome coronavirus 2] | QHR84449.1 | ✔ | ✔ | ✔ | ✔ | ✔ | ✔ |
| surface glycoprotein [Severe acute respiratory syndrome coronavirus 2] | QHZ00379.1 | ✔ | ✔ | ✔ | ✔ | ✔ | ✔ |
| surface glycoprotein [Severe acute respiratory syndrome coronavirus 2] | QIC53204.1 | ✔ | ✔ | ✔ | ✔ | ✔ | ✔ |
| surface glycoprotein [Severe acute respiratory syndrome coronavirus 2] | QHU79173.1 | ✔ | ✔ | ✔ | ✔ | ✔ | ✔ |
| spike glycoprotein [Bat coronavirus RaTG13] | QHR63300.2 | ✔ |  | ✔ | ✔ | ✔ |  |
| spike protein [Rhinolophus affinis coronavirus] | AHX37569.1 |  |  | ✔ |  |  |  |
| spike protein [Rhinolophus affinis coronavirus] | AHX37558.1 |  |  | ✔ |  |  |  |
| spike protein [Bat SARS-like coronavirus] | AVP78031.1 |  |  | ✔ |  |  |  |
| spike protein [Bat SARS-like coronavirus] | AVP78042.1 |  |  | ✔ |  |  |  |
| spike protein [Bat SARS-like coronavirus] | ATO98181.1 |  |  | ✔ |  |  |  |
| spike glycoprotein [Bat SARS coronavirus HKU3-8] | ADE34766.1 |  |  | ✔ |  |  |  |
| spike glycoprotein [Bat SARS-like coronavirus] | AID16716.1 |  |  | ✔ |  |  |  |
| spike glycoprotein [Coronavirus BtRs-BetaCoV/YN2018A] | QDF43820.1 |  |  | ✔ |  |  |  |
| spike glycoprotein [Bat SARS coronavirus HKU3-12] | ADE34812.1 |  |  | ✔ |  |  |  |
| spike glycoprotein [Bat SARS coronavirus HKU3-7] | ADE34755.1 |  |  | ✔ |  |  |  |
| spike glycoprotein [BtRs-BetaCoV/GX2013] | AIA62320.1 |  |  | ✔ |  |  |  |
| spike protein [Bat SARS Cov Rs806/2006] | ACU31051.1 |  |  | ✔ |  |  |  |
| spike protein [Bat SARS-like coronavirus] | ATO98169.1 |  |  | ✔ |  |  |  |
| spike protein [Bat SARS-like coronavirus] | ATO98120.1 |  |  | ✔ |  |  |  |
| spike protein [Bat SARS-like coronavirus] | ATO98108.1 |  |  | ✔ |  |  |  |
| spike glycoprotein [Coronavirus BtRs-BetaCoV/YN2018D] | QDF43835.1 |  |  | ✔ |  |  |  |
| spike glycoprotein [Bat SARS-like coronavirus YNLF_34C] | AKZ19087.1 |  |  | ✔ |  |  |  |
| spike protein [Bat SARS CoV Rm1/2004] | ABD75332.1 |  |  | ✔ |  |  |  |
| spike glycoprotein [BtRf-BetaCoV/HeN2013] | AIA62339.1 |  |  | ✔ |  |  |  |
| spike glycoprotein [BtRf-BetaCoV/HeB2013] | AIA62290.1 |  |  | ✔ |  |  |  |
| protein S2'; Flags: Precursor [Bat SARS CoV Rp3/2004] | Q3I5J5.1 |  |  |  | ✔ |  |  |
| spike protein [Bat SARS-like coronavirus] | ATO98193.1 |  |  | ✔ |  |  |  |
| spike glycoprotein [Coronavirus BtRs-BetaCoV/YN2018C] | QDF43830.1 |  |  | ✔ |  |  |  |
| spike glycoprotein [Coronavirus BtRl-BetaCoV/SC2018] | QDF43815.1 |  |  | ✔ |  |  |  |
| spike protein [SARS coronavirus Rs_672/2006] | ACU31032.1 |  |  | ✔ |  |  |  |
| spike protein [Bat CoV 273/2005] | ABG47060.1 |  |  | ✔ |  |  |  |
| spike glycoprotein [BtRf-BetaCoV/HuB2013] | AIA62340.1 |  |  | ✔ |  |  |  |
| spike glycoprotein [BtRs-BetaCoV/HuB2013] | AIA62310.1 |  |  |  | ✔ | ✔ |  |
| Spike protein S2'; Flags: Precursor [Bat CoV 279/2005] | Q0Q475.1 |  |  |  | ✔ |  |  |
| spike protein [Bat SARS CoV Rf1/2004] | ABD75323.1 |  |  | ✔ |  |  |  |
| spike glycoprotein [Bat SARS-like coronavirus YNLF_31C] | AKZ19076.1 |  |  | ✔ |  |  |  |
| spike glycoprotein [Bat coronavirus] | ARI44809.1 |  |  | ✔ |  |  |  |
| spike protein [Bat coronavirus Cp/Yunnan2011] | AGC74176.1 |  |  | ✔ |  |  |  |
| spike protein [Bat coronavirus Rp/Shaanxi2011] | AGC74165.1 |  |  | ✔ |  |  |  |
| spike glycoprotein [BtRf-BetaCoV/SX2013] | AIA62300.1 |  |  | ✔ |  |  |  |
| spike glycoprotein [BtRf-BetaCoV/JL2012] | AIA62277.1 |  |  | ✔ |  |  |  |
| spike protein [Bat coronavirus] | ANH10613.1 |  |  | ✔ |  |  |  |
| spike protein [Severe acute respiratory syndrome-related coronavirus] | ARO76382.1 |  |  | ✔ |  |  |  |
| spike protein [Bat coronavirus] | ANA96027.1 |  |  | ✔ |  |  |  |
| spike glycoprotein [Bat coronavirus] | ASO66810.1 |  |  | ✔ |  |  |  |
| spike protein [Bat SARS-like coronavirus] | ATO98145.1 |  |  | ✔ |  |  |  |
| spike glycoprotein [Bat coronavirus] | ARI44799.1 |  |  | ✔ |  |  |  |
| spike glycoprotein [BtRs-BetaCoV/YN2013] | AIA62330.1 |  |  | ✔ |  |  |  |
| spike protein [Bat coronavirus] | ANA96090.1 |  |  | ✔ |  |  |  |

## Supplementary Table S4: Table presenting statistics of interaction of all three vaccine constructs with HLA superfamily alleles. Here a lower HADDOCK score indicates the higher strength of interaction between the proteins.

|  | **Vaccine1** | | | **Vaccine2** | | | **Vaccine3** | | |
| --- | --- | --- | --- | --- | --- | --- | --- | --- | --- |
|  | **HLA A** | **HLA B** | **HLA DRB1** | **HLA A** | **HLA B** | **HLA DRB1** | **HLA A** | **HLA B** | **HLA DRB1** |
| **HADDOCK score** | -263.1 +/- 3.2 | -242.1 +/- 1.3 | -279.2 +/- 3.7 | -273.9 +/- 3.4 | -249.3 +/- 4.5 | -269.9 +/- 4.5 | -190.9 +/- 2.6 | -205.4 +/- 3.2 | -191.8 +/- 4.9 |
| **Cluster size** | 20 | 20 | 20 | 20 | 20 | 20 | 20 | 20 | 20 |
| **RMSD from the overall lowest-energy structure** | 0.3 +/- 0.2 | 0.3 +/- 0.2 | 0.3 +/- 0.2 | 0.3 +/- 0.2 | 0.3 +/- 0.2 | 0.3 +/- 0.2 | 0.3 +/- 0.2 | 0.3 +/- 0.2 | 0.3 +/- 0.2 |
| **Van der Waals energy** | -147.7 +/- 5.8 | -150.9 +/- 4.3 | -181.4 +/- 0.9 | -155.3 +/- 5.2 | -130.1 +/- 7.0 | -155.8 +/- 6.0 | -111.1 +/- 1.5 | -127.3 +/- 5.0 | -118.6 +/- 4.5 |
| **Electrostatic energy** | -387.1 +/- 19.0 | -367.4 +/- 13.0 | -334.1 +/- 23.8 | -547.8 +/- 16.8 | -658.4 +/- 32.0 | -545.9 +/- 6.4 | -396.9 +/- 10.1 | -371.5 +/- 33.2 | -501.5 +/- 17.5 |
| **Desolvation energy** | -38.0 +/- 5.3 | -17.7 +/- 5.7 | -31.0 +/- 5.2 | -9.0 +/- 1.6 | 12.4 +/- 6.5 | -4.9 +/- 7.2 | -0.5 +/- 3.5 | -3.8 +/- 1.8 | 27.0 +/- 5.1 |
| **Restraints violation energy** | 0.2 +/- 0.06 | 0.1 +/- 0.04 | 0.1 +/- 0.17 | 0.2 +/- 0.09 | 0.1 +/- 0.04 | 0.1 +/- 0.02 | 0.1 +/- 0.04 | 0.2 +/- 0.07 | 0.1 +/- 0.06 |
| **Buried Surface Area** | 4075.6 +/- 48.5 | 4134.6 +/- 62.6 | 4937.1 +/- 33.7 | 4548.7 +/- 83.7 | 3858.1 +/- 45.7 | 4325.5 +/- 38.6 | 3074.2 +/- 26.3 | 3076.2 +/- 7.9 | 3649.3 +/- 18.4 |
